# A novel multi-scale and fine-grained network for large choroidal vessels segmentation in OCT

**DOI:** 10.3389/fcell.2025.1508358

**Published:** 2025-01-28

**Authors:** Wei Huang, Qifeng Yan, Lei Mou, Yitian Zhao, Wei Chen

**Affiliations:** ^1^ School of Biomedical Engineering, Hainan University, Haikou, China; ^2^ Laboratory of Advanced Theranostic Materials and Technology, Ningbo Institute of Materials Technology and Engineering, Chinese Academy of Sciences, Ningbo, China; ^3^ Ningbo Key Laboratory of Medical Research on Blinding Eye Diseases, Ningbo Eye Institute, Ningbo Eye Hospital, Wenzhou Medical University, Ningbo, China; ^4^ National Clinical Research Center for Ocular Diseases, Eye Hospital, Wenzhou Medical University, Wenzhou, China

**Keywords:** optical coherence tomography, choroid, segmentation algorithm, 3D reconstruction, feature analysis

## Abstract

Accurate segmentation of large choroidal vessels using optical coherence tomography (OCT) images enables unprecedented quantitative analysis to understand choroidal diseases. In this paper, we propose a novel multi-scale and fine-grained network called MFGNet. Since choroidal vessels are small targets, long-range dependencies need to be considered, therefore, we developed a two-branch fine-grained feature extraction module that can mix the long-range information extracted by TransFormer with the local information extracted by convolution in parallel, introducing information exchange between the two branches. To address the problem of low contrast and blurred boundaries of choroidal vessels in OCT images, we developed a large kernel and multi-scale attention module, which can improve the features of the target area through multi-scale convolution kernels, channel mixing and feature refinement. We quantitatively evaluated the MFGNet on 800 OCT images with large choroidal vessels manually annotated. The experimental results show that the proposed method has the best performance compared to the most advanced segmentation networks currently available. It is noteworthy that the large choroidal vessels were reconstructed in three dimensions (3D) based on the segmentation results and several 3D morphological parameters were calculated. The statistical analysis of these parameters revealed significant differences between the healthy control group and the high myopia group, thereby confirming the value of the proposed work in facilitating subsequent understanding of the disease and clinical decision-making.

## 1 Introduction

As the main blood supply structure to the outer retinal layer and the anterior nerve of the cribriform plate, the choroid is the most densely packed part of the body’s blood vessels. It provides nutrients to the retina to maintain normal light-sensitive function while secreting growth factors and regulating body temperature (Nickla and Wallman, 2010). The vascular structure of the choroid is very complex and can be divided into three sublayers: the capillary layer near the retina (choriocapillaris), the middle vascular layer (Sattler’s layer), and the large vascular layer near the sclera (Haller’s layer).

Optical coherence tomography (OCT) represents the most widely utilised innovative technology in recent years for the clinical diagnosis of retinal diseases. It is a non-contact, non-invasive, high-resolution imaging technique that is capable of displaying clear structural maps of the choroid and its sublayers of blood vessels (Huang et al., 1991). At present, the analysis of the choroid in OCT images is a widely employed methodology for investigating the aetiology of associated pathologies.

Clinical studies have shown that changes in the vascular morphology of the choroidal Haller layer are closely related to the pathological processes of many ocular diseases. For example, in patients with high myopia (HM), the thickness of the Haller layer blood vessels may become thinner; Patients with high myopia may also experience changes in Haller vessel density (Wang et al., 2015). In patients with intermediate-stage age-related macular degeneration (AMD), Haller’s layer vascular thickness is reduced; The thickness variation of the Haller layer may be related to the development of AMD (Torzicky et al., 2012). In patients with diabetes retinopathy, the diameter of the blood vessels in the Haller layer changes; The changes in the diameter of Haller’s layer blood vessels may reflect changes in the choroidal microcirculation (Gulshan et al., 2016). In conclusion, further research into the changes in the blood vessels of Haller’s layer is of potential value in understanding, screening and diagnosing these diseases.

Recently, total choroidal thickness is considered an important parameter for diagnosis because it is highly related to several pathological conditions (Zhang et al., 2012; Garvin et al., 2008; Hu et al., 2013; Li et al., 2005; Sui et al., 2017; He et al., 2021; Zhang et al., 2020; Chai et al., 2020; Yan et al., 2023; Wu et al., 2022). With the continuous development of OCT imaging technology, imaging within the choroid has become clearer, and many studies have focused on the relationship between changes in choroidal sublayer thickness and diseases. However, this method does not fully reflect the characteristics of the choroid and can occasionally result in an invalid diagnosis. For instance, it has been reported that glaucoma can lead to either thinning or thickening of the choroid at different stages (Mwanza et al., 2011). One hypothesis that can explain this inconsistency is that different changes occur in the vascular system of each sublayer of the choroid (Yin et al., 1997). In addition, the overall and sublayer thickness changes of the choroid are caused by the dilation or atrophy of the blood vessels within the choroid. Previous studies have shown that choroidal blood flow is reduced in patients with high myopia, AMD and other diseases (Akyol et al., 1996). To better explain the properties of choroidal vessels, Agrawal et al. (Agrawal et al., 2016b) proposed a new quantitative parameter, choroidal vascular index (CVI), and reported the application of CVI in a variety of ocular diseases in many studies, including diabetic retinopathy (Agrawal et al., 2016d), central serous chorioretinopathy (Agrawal et al., 2016a) and polypoid chorioretinopathy (Agrawal et al., 2016c). Therefore, automatic segmentation of the large choroidal vessels is beneficial to efficiently observe changes in choroidal vessels and blood flow in more patients. By focusing on the vascular changes within the choroid and visualizing them in 3D, we can more directly examine the relationship between morphological changes and disease.

In the last decade, some studies focused on segmentation of choroidal macrovessels on OCT images. These methods can be broadly classified into traditional hand-based approaches (Duan et al., 2013; Kajić et al., 2013; Zhang et al., 2012; Srinath et al., 2014; Nasar et al., 2019) and deep learning-based approaches (Khaing et al., 2021; Zheng et al., 2021; Ronneberger et al., 2015; Zhu et al., 2022a; Liu et al., 2019; Huang et al., 2023). Traditional methods can only demonstrate the visual impact of large choroidal vessels segmentation, lacking a gold standard for quantitative assessment through manual annotation. Deep learning methods have inductive biases such as local correlation and translation during the calculation process and lack global modeling capabilities, making it difficult to achieve high-precision blood vessel segmentation. Moreover, there is a notable absence of disease correlation analysis based on the comprehensive three-dimensional morphological features of the choroidal vasculature, which could facilitate a deeper understanding of associated disease progression and inform clinical decision-making.

Consequently, we present a novel deep neural network, namely, a multiscale and fine-grained network (MFGNet), for automated segmentation of choroidal vessels in OCT images. Specifically, MFGNet uses U-Net as the backbone. Since choroidal vessels are small targets, long-range dependencies need to be considered, therefore, we developed a two-branch fine-grained feature extraction module that can mix the long-range information extracted by TransFormer with the local information extracted by convolution in parallel, introducing information exchange between the two branches. To address the problem of low contrast and blurred boundaries of choroidal vessels in OCT images, we developed a large kernel and multi-scale attention module, which can improve the features of the target area through multi-scale convolution kernels, channel mixing and feature refinement.

Our contributions to the work are summarized as follows:

• In response to the low contrast and blurred boundaries of the choroidal vessels in OCT images, we developed the Large Kernel and Multi Scale Attention (LKSA) module. It achieves precise segmentation of choroidal vessels by using large kernels and multiscale perception of changes in vascular structural information.

• We developed a dual-branch fine-grained feature extraction (DFG) module to address the small and widely distributed choroidal vessels. The DFG module uses a parallel design to mix the remote information extracted by TransFormer with the local information extracted by convolution and introduce information exchange between the two branches.

• Based on the results of the segmentation process, a comprehensive three-dimensional reconstruction of the choroidal vasculature was performed. A statistical analysis of the morphological parameters derived from the reconstructed three-dimensional vascular network of clinical data indicates that the proposed methodology can facilitate subsequent disease understanding and clinical decision-making.

## 2 Related works

Below we will briefly discuss the associated work on the segmentation of large choroidal vessels.

Traditional methods for segmenting large blood vessels in the choroid typically require prior knowledge and the use of pre- or post-processing to denoise the input image to improve segmentation results. Duan et al. (2013) and Kajić et al. (2013) applied a multiscale adaptive thresholding method to segment large blood vessels in the choroid. After segmenting the choroidal vessels, it calculates the depth-related signal-to-noise ratio based on the ideal vascular response based on registration and multi-scale filtering and performs a three-dimensional reconstruction of the vessels to obtain three-dimensional statistical data. Zhang et al. (2012) proposed to use a 3D tube model to fit the large blood vessels of the choroid, and then optimize the segmented choroidal blood vessel boundaries through multi-scale Hessian filters and threshold processing results. Likewise, 3D reconstruction of large blood vessels enables a more intuitive representation of vascular information. Srinath et al. (2014) proposed a method in which the upper and lower boundaries of the choroid are first identified and then the blood vessels are iteratively segmented by applying level sets to the detected choroidal layer. By extracting the choroid, they can improve the detection of blood vessels and their subsequent segmentation. Nasar et al. (2019) chose centerline-based TEASAR estimation to calculate the normals and cross sections of blood vessels. They then iteratively moved the centerline to the centroid of the existing cross-section and smoothed it using a three-dimensional tensor until the changes between the two iterations were not significant. However, all of the above methods only show the visual effects of large vessel segmentation of the choroid, lacking a gold standard for quantitative evaluation through manual annotation. Therefore, determining accuracy and reproducibility presents certain challenges.

As it is the most well-known technology in the field of medical image segmentation, some studies have attempted to apply deep learning methods to the segmentation of large blood vessels in the choroid of OCT images. In a seminal contribution to the field, Khaing et al. (2021) developed an end-to-end architectural framework, designated as ChoroidNet, which integrates convolutional neural networks (CNN) and advanced convolutions for the efficient segmentation of the choroidal layer and its associated vasculature. Zheng et al. (2021) achieved the segmentation and quantification of choroidal vessels in OCT images by adding residual modules to the U-Net network (Ronneberger et al., 2015). However, because it is a gold standard image, it cannot classify the morphology of vascular structures well, which increases the difficulty of subsequent vascular reconstruction work. Zhu et al. (2022a) introduced a multi-task learning network, designated as CUNet, which employs a shared encoder and three decoders for the joint segmentation of choroidal layers and vessels. In a recent study, Liu et al. (2019) proposed an end-to-end model for training and testing large blood vessel segmentation in the choroid, which does not require any pre- or post-processing steps. This method is appropriate for the segmentation of large blood vessels in the choroid, which typically comprise a multitude of areas with intricate patterns that are challenging to analyse manually. However, this method requires a substantial quantity of annotated data, which presents a challenge in achieving accurate segmentation on small datasets. Huang et al. (2023) put forth a 3D convolutional neural network (CNN) approach, designated as SAN, which employs anisotropic downsampling and upsampling operations to construct a 3D U-shaped network backbone. Additionally, it incorporates an auxiliary output branch to predict a novel choroidal vessel shape representation, generated through the application of labels.

## 3 Proposed method

In this section, we present the overall structure of the proposed multi-scale and fine-grained choroidal vessel segmentation network (MFGNet). MFGNet consists of two key module designs: (1) using a large kernel and multi-scale attention module (LKSA module) in the low-level (stem) phase; (2) In the downsampling phase, a two-branch fine-grained feature extraction module (DFG module) with convolution and TransFormer is used. They extract fine-grained features through large-nuclei fusion and multi-scale perception of structural information changes in blood vessels, as well as global and local bidirectional fusion.

**FIGURE 1:**
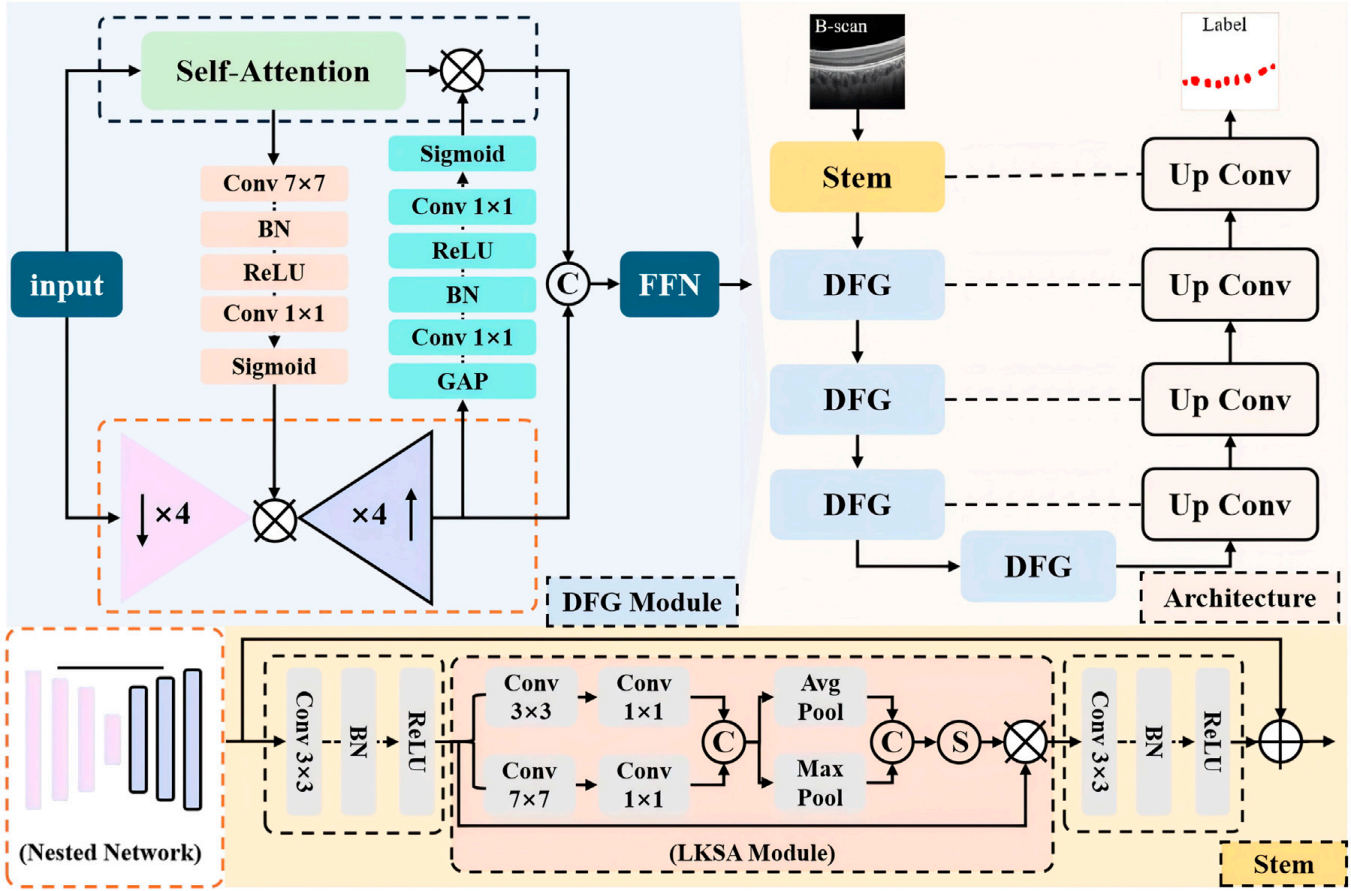
The overall structure diagram of our MFGNet.

The overall structure is shown in Figure 1, with MFGNet using U-Net as the backbone. It includes a stem layer and four downsampling layers for progressive feature extraction, while using corresponding upsampling blocks for feature reconstruction. Below, the Stem layer contains two Conv-BN-ReLU units and LKSA module in the middle. And Each of the remaining encoder layers contains two DFG modules. In the decoder stage, the feature reconstruction layer includes bilinear interpolation for upsampling and two Conv-BN-ReLU structures for feature reconstruction. We continued U-Net’s skip connections to make it easier to merge feature information. The specific information for the LKSA module and DFG module is as follows.

### 3.1 Large kernel and multi-scale attention module

In the low-level stage of neural networks, the network tends to extract more detailed information such as contours for feature extraction. On OCT, the contrast of the choroidal macrovessels is low and the boundaries are blurred. For this reason, we developed a large kernel and multi-scale attention module to extract local and contour information of blood vessels to address this problem. The specific details of the LKSA module are shown in the orange dashed box in Figure 1. The LKSA module initially includes two Depth-wise convolutions for multi-scale feature extraction between individual feature channels with convolution kernels of 3 × 3 and 7 × 7, respectively. Then two 1 × 1 convolutions merge this feature information in the channel dimension. We concatenate the feature maps in the channel dimension and perform mean pooling and max pooling. The pooled feature map keeps its spatial dimension unchanged while its channel dimension is compressed to 1. The compressed pooled feature maps were concatenated and mapped using the Sigmoid function to obtain multi-scale attention weights. We perform feature correction on the input feature map using attention weights. The advantage of this operation is to improve the features of the target area through multi-scale convolution kernels, channel mixing and feature refinement. The formula is as follows:

$$AvgPool_{w}=AvgPool\left(Concat\left(Conv_{1\times 1}\left(Conv_{3\times 3}\left(X\right)\right),Conv_{1\times 1}\left(Conv_{7\times 7}\left(X\right)\right)\right)\right)$$ (1)

$$MaxPool_{w}=MaxPool\left(Concat\left(Conv_{1\times 1}\left(Conv_{3\times 3}\left(X\right)\right),Conv_{1\times 1}\left(Conv_{7\times 7}\left(X\right)\right)\right)\right)$$ (2)

$$Attention=Sigmoid\left(Concat\left(AvgPool_{w}+MaxPool_{w}\right)\right)$$ (3)

Out=X+X×Attention (4)

where X represents the input feature map, and $AvgPool_{w}$ and $MaxPool_{w}$ refer to the weight maps obtained via AvgPool and MaxPool.

### 3.2 Dual-branch fine-grained feature extraction module

Due to the small and widely distributed blood vessels in the choroid, it is necessary for the network to extract both local information and long-range dependency information to accurately identify the target area. We have developed a novel dual-branch fine-grained feature extraction module (DFG). The DFG module uses a parallel design to mix the remote information extracted by TransFormer with the local information extracted by convolution and introduce information exchange between the two branches. TransFormer’s self-attention mechanism can effectively model long-range information, but its ability to model channel information is limited due to information flattening during the calculation process. We introduced a nested network in the DFG module, which includes four downsampling blocks and the corresponding upsampling blocks. Simply put, this is a simplified version of the U-shaped structure. Each convolution block contains a Conv-BN-ReLU structure. Although this nested network stack can model fine-grained feature information, it lacks the ability to model global spatial information due to the exclusive use of convolution. In summary, we have introduced information exchange between the two feature extraction branches.

First, we use a SE block to extract channel attention weights at the output layer of the nested network to compensate for the shortcomings of TransFormer in modeling channel information. Second, a spatial attention block is introduced into the bottleneck layer of the nested network. This spatial attention block includes 7 × 7 convolution, BN, ReLU, 1 × 1 convolution and sigmoid function, where the stride of 7 × 7 convolution is 4 to take into account the spatial dimension of the nested network bottleneck layer. Finally, we link the self-attention gained by interacting with feature information to the output of the nested network. The Feed forward network (FFN) layer is used to effectively fuse the outputs of double branches, consisting of a fully connected layer, a 3 × 3 Depth-wise convolution, GELU, and a second fully connected layer.

### 3.3 Loss function

To overcome the problem of blurred boundaries of large choroidal vessels, we use a mixed cross-entropy $\left(L_{CE}\right)$ and Dice $\left(L_{\mathrm{DICE}}\right)$ loss function, which can better deal with the ambiguity and class inequality of the target area in the image. Specifically as follows:

$$L=\alpha \cdot L_{CE}+\left(1-\alpha \right)\cdot L_{\mathrm{DICE}}$$ (5)

where α is set as 0.5 in our task.

## 4 Experimental settings

### 4.1 Dataset

In this work, The dataset we used is the publicly available dataset OCHID in our previous work. The introduction of the dataset can refer to (Yan et al., 2024). The code link can be found on https://github.com/iMED-Lab/MFGNet.

### 4.2 Implementation

The network architecture was implemented using the PyTorch library, which is a Python library for machine learning and artificial intelligence. All experiments were conducted on a workstation equipped with a single NVIDIA GeForce GTX 3090 GPU with 24 GB of memory. The proposed network was trained for 400 epochs, with the following hyperparameters set: The Adam optimisation method was employed, with an initial learning rate of 0.0007 and a batch size of 4. In order to facilitate comparison, the training strategy outlined in the original paper was adopted for all other methods.

### 4.3 Evaluation metrics

We use Dice , IoU and Pre as evaluation metrics for large choroidal vessel segmentation as follows:

$$Dice=\frac{2\cdot TP}{2\cdot TP+FP+FN}$$ (6)

$$IoU=\frac{TP}{TP+FP+FN}$$ (7)

$$Pre=\frac{TP}{2\cdot TP+FP}$$ (8)

where TP , FP and FN represent true positives, false positives and false negatives, respectively.

## 5 Experimental results

To validate the effectiveness of the proposed method in segmenting large choroidal vessels, we conducted a comprehensive comparative study. For choroidal vessel segmentation, we selected 11 CNN and Transformer-based models with generalization capabilities: U-Net (Ronneberger et al., 2015), CE-Net (Gu et al., 2019), Seg-Net (Badrinarayanan et al., 2017), ResUNet (Zheng et al., 2021), ChoroidNet (Khaing et al., 2021), TransUNet (Chen et al., 2021), SegFormer (Xie et al., 2021), PoolFormer (Yu et al., 2022), LawinFormer (Kajić et al., 2013), Swin-Transformer (Liu et al., 2021) and CLA-net (Yan et al., 2024).

### 5.1 Qualitative results

**FIGURE 2:**
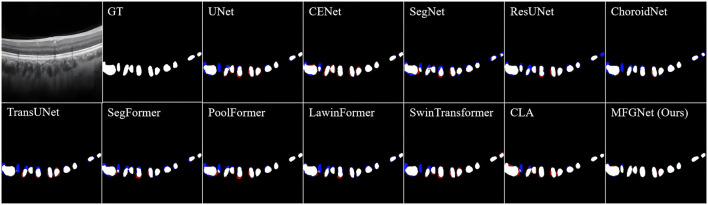
The large choroidal vessel segmentation results by different methods. Note, the red denotes over-segmentation, and blue denotes under-segmentation.

Figure 2 shows the visualization results of various segmentation networks in the segmentation stage of large choroidal vessels. The red area in the figure is the over-segmentation area and the blue area is the under-segmentation area. As observed in the ground truth, the spatial distribution of large vessel morphology in the Haller layer is unpredictable. Therefore, the segmentation results based on CNN or Transformer models all indicate under-segmentation or over-segmentation. Compared with the real label map, MFGNet’s segmentation results do not have excessive segmentation, which ensures the authenticity of segmentation, and also do not have large areas of insufficient segmentation, which ensures the accuracy of subsequent feature analysis. Overall, MFGNet achieved the best performance in large vessel segmentation, which could further facilitate 3D reconstruction of choroidal vessels.

### 5.2 Quantitative results

Table 1 shows the quantitative results of different methods in segmenting large blood vessels in the choroid. It can be observed that MFGNet has the best performance in Dice, IoU and Pre evaluation metrics. Taking the Dice metric as an example, MFGNet achieved a segmentation result of 76.2%, which is 2.40% higher than U-Net. The main advantage of MFGNet in large vessel segmentation tasks is the design of a large-kernel and multi-scale attention module at the low-level, which focuses more on feature extraction of detailed information such as contours. From a quantitative perspective, the accuracy and suitability of MFGNet for large choroidal vessels segmentation were also validated.

**TABLE 1:** Large choroidal vessel segmentation results by different methods.

Methods	Dice ↑	IoU ↑	Pre ↑
U-Net	0.738 ± 0.055	0.588 ± 0.067	0.694 ± 0.884
CE-Net	0.749 ± 0.060	0.602 ± 0.075	0.694 ± 0.084
Seg-Net	0.729 ± 0.054	0.576 ± 0.065	0.665 ± 0.096
ResUNet	0.736 ± 0.058	0.586 ± 0.071	0.691 ± 0.090
ChoroidNet	0.731 ± 0.063	0.580 ± 0.076	0.695 ± 0.096
TransUNet	0.750 ± 0.054	0.603 ± 0.068	0.708 ± 0.089
SegFormer	0.744 ± 0.052	0.596 ± 0.065	0.706 ± 0.088
PoolFormer	0.733 ± 0.056	0.581 ± 0.068	0.674 ± 0.081
LawinFormer	0.737 ± 0.056	0.586 ± 0.069	0.670 ± 0.090
Swin-Transformer	0.745 ± 0.060	0.596 ± 0.062	0.704 ± 0.077
CLA-net	0.760 ± 0.051	0.616 ± 0.065	0.729 ± 0.080
MFGNet	**0.762** ± **0.060**	**0.619** ± **0.079**	**0.751** ± **0.090**

The values in bold represent the best of all the comparative experimental results.

### 5.3 Ablation studies

In order to better understand the role of LKSA and DFG modules in MFGNet, a series of relevant ablation experiments were conducted for verification in this section, as shown in Table 2. MFGNet (LKSA) added to U-Net with LKSA module showed an improvement of 1.9% on Dice, 2.4% on IoU, and 6.7% on Pre compared to U-Net. MFGNet (DFG) with added DFG module increased Dice by 2.0%, IoU by 2.5% and Pre by 3.5% compared to U-net. Compared to U-net, MFGNet (LKSA + DFG) increased Dice by 2.4%, IoU by 3.1%, and Pre by 5.7%. The above experimental results show that the LKSA and DFG modules are effective and can significantly improve the algorithm performance.

**TABLE 2:** Ablation studies for large choroidal vessel segmentation.

Methods	Dice ↑	IoU ↑	Pre ↑
U-Net	0.738 ± 0.055	0.588 ± 0.067	0.694 ± 0.884
MFGNet (LKSA)	0.757 ± 0.060	0.612 ± 0.073	**0.761** ± **0.074**
MFGNet (DFG)	0.758 ± 0.060	0.613 ± 0.074	0.729 ± 0.089
MFGNet	**0.762** ± **0.060**	**0.619** ± **0.079**	**0.751** ± **0.090**

The values in bold represent the best of all the comparative experimental results.

## 6 Clinical applications

The thickness characteristics of the choroid have become an important observation factor for the diagnosis and treatment of high myopia in clinical practice, but information about the thickness alone is not enough to fully reflect the changes in the overall morphology of the choroidal structure. The most common vascular structural features in the choroid have not received much attention due to their difficulty in extraction. In addition, most related studies are based on two-dimensional morphological indicators (Li et al., 2021; Zhu et al., 2022b) without in-depth analysis of three-dimensional features. For three-dimensional vascular structures and ocular structures, it is difficult to analyze them exclusively from a two-dimensional perspective to provide guarantees for clinical research and disease diagnosis and treatment. Therefore, this article aims to study the correlation between the three-dimensional vascular characteristics of the choroid and high myopia from the perspective of three-dimensional structure, reconstruct the large blood vessels of the choroid in three dimensions, and analyze the correlation between multiple characteristics such as three-dimensional vascular density, three-dimensional analysis dimension as well as three-dimensional vascular curvature and high myopia, and provide new ideas for understanding the pathogenesis of high myopia and subsequent prevention and treatment.

This section used 50 OCT volume data collected by the Spectrails OCT2 device from Heidelberg, which included 18 highly myopic patients and 32 healthy controls. The subjects were aged between 20 and 30 years old, and all subjects collected monocular images. Each volume data contained 512 B-Scan images with a resolution of 378 × 379 pixels, covering an area of 3 × 3 × 2 $mm^{3}$ centered on the fovea centralis.

### 6.1 3D reconstruction of large choroidal vessels

The three-dimensional reconstruction of large blood vessels in the choroid is based on the proposed MFGNet for segmentation and extraction of large blood vessel structures from the dataset. Due to the scattered location and irregular shape of large blood vessels, it is not possible to use the same reconstruction method as the layered structure, which may result in many points being eliminated as outliers. In this chapter, the 2D segmentation result image is saved as a 3D binary image represented by a 0–1 value array. A mesh generation method (Si and Gärtner, 2005) is applied to create a triangular mesh corresponding to the voxel resolution with boundaries. It is then applied to the resampled surface mesh based on the specified surface mesh density. In the 3D reconstruction of large blood vessels, the surface mesh density is set to 0.8.

In the process of 3D surface reconstruction, various topological defects may exist, such as: E.g., isolated vertices, repeated triangles, non-manifold vertices, *etc.* (Fang and Boas, 2009). Therefore, in this chapter, to ensure the accuracy of the final reconstruction effect, mesh inspection and repair were carried out before the final reconstruction. For surface smoothing and optimization, the Laplacian operator is used, which acts directly on the topological domain and produces smooth grids in several iterations. His smoothing method is specifically presented as follows:

$$p^{\prime }=p+\frac{\lambda }{n}\overset{n-1}{\underset{i=0}{\sum }}\left(q_{i}-p\right)$$ (9)

Where, λ represents the weighting factor, n the number of iterations, p the vertex before smoothing, $p^{\prime }$ the new position after smoothing and $q_{i}$ the neighboring points of the original vertex. For three-dimensional reconstruction of large choroidal vessels, λ is set to 0.9 and n to 10.

**FIGURE 3:**
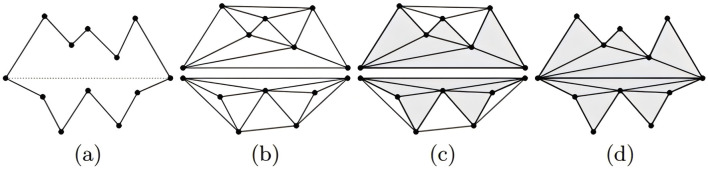
Visual illustration of mesh reconstruction at the two-dimensional level (Si and Gärtner, 2005). **(A)** Two initial cavities separated by a constrained segment; **(B)** construction of Delaunay triangles; **(C)** marking the triangles as “inside” or “outside”; **(D)** removing the “outside” triangles.

**FIGURE 4:**
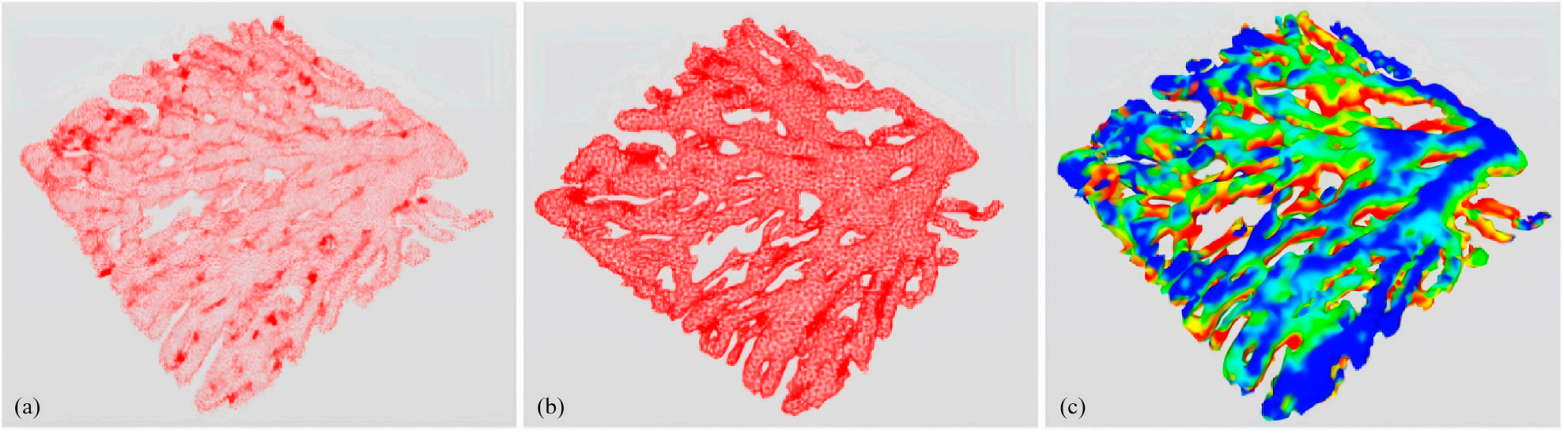
Three-dimensional reconstruction of choroidal major vessels. **(A)** 3D point cloud; **(B)** 3D mesh; **(C)** 3D vascular structure rendered with curvature size.

Finally, using the subregion labeling algorithm to generate internal points, a 3D multi-region tetrahedral network with a certain grid density is created, resulting in the 3D reconstruction of large blood vessels. Figure 3 illustrates the reconstruction principle from a two-dimensional perspective. The final three-dimensional reconstruction of the macrovascular structure of the choroid is shown in Figure 4.

### 6.2 Feature extraction and analysis

#### 6.2.1 Three-dimensional fractal dimension (3D-FD)

The three-dimensional fractal dimension is used to measure the spatial complexity of three-dimensional fractal shapes and the symmetry and irregularity of vessel fractals. In general, the larger the three-dimensional fractal dimension, the more complex the fractal is, indicating that the fractal has more asymmetry and randomness. It can be expressed as: $3D-FD=lim\frac{logN_{r}}{log\left(1/r\right)}$ , where r is the length of the box’s side, N indicates the total number of boxes, which cover all vessel regions.

#### 6.2.2 Three-dimensional vascular density (3D-VD)

Three-dimensional vascular density refers to the density of the vascular network in three-dimensional space, which can measure the number and distribution of blood vessels and is used to evaluate the health status of the vascular system and the subsequent detection of vascular diseases. The three-dimensional reconstruction results of large blood vessels based on the choroid are calculated by calculating the length or quantity of blood vessels in each unit volume. The study of three-dimensional vascular density in the choroid is of great importance for improving eye health and preventing eye diseases.

#### 6.2.3 Three-dimensional vascular curvature (3D-VC)

Three-dimensional vascular curvature refers to the degree of curvature of blood vessels in three-dimensional space, which can measure the shape and morphology of blood vessels, and can also be used to assess the health status of blood vessels and diagnose vascular diseases. The three-dimensional vascular curvature of the choroid is determined by analyzing the three-dimensional shape and curvature of the blood vessels.

The quantitative analysis results of the three-dimensional characteristics of large blood vessels in the choroid are shown in Table 3. According to Table 3, there is no significant difference in the three-dimensional vessel curvature characteristics between the healthy population and the highly myopic population, the *t*-test value of the characteristic indicators for both groups is 0.693. As far as three-dimensional fractal dimension and three-dimensional vascular density are concerned, there is a statistical difference between the healthy population and the severely myopic population. The average three-dimensional vascular density of the healthy population is 12.0294 ± 3.6173, while the average three-dimensional fractal dimension of the highly myopic population is 9.1604 ± 5.0237. After testing and analyzing the two types of data, the value is 0.023, indicating that the difference between the two is significant.

**TABLE 3:** Comparison of 3D characteristics of choroidal large vessels.

Subject	Volume	3D-FD	3D-VD	3D-VC
Healthy Control	32	2.2506 ± 0.0718	12.0294 ± 3.6173	0.1959 ± 0.0046
High Myopia	18	2.1813 ± 0.1255	9.1604 ± 5.0237	0.1949 ± 0.0092
		P=0.041 < 0.05	P=0.023 < 0.05	P = 0.693

There are statistical differences in three-dimensional fractal dimension and three-dimensional vascular density between highly myopic individuals and healthy individuals. The main reason for this is that the thickness of the choroid and retina is thinner in highly myopic people, and the choroidal blood vessels are in a certain degree of atrophy, which reduces the complexity of the blood vessels in the entire three-dimensional space three-dimensional fractal dimension. Similarly, as the area occupied by blood vessels decreases throughout the choroidal structure, the three-dimensional vascular density also decreases in some sense. However, the curvature of three-dimensional blood vessels is mainly influenced by vascular elasticity, so vascular morphological curvature is more common in diseases such as hypertension, which involve changes in the blood vessels themselves; However, in highly myopic people there are no changes in vascular pressure and vascular elasticity, so their vascular curvature does not show any significant differences compared to healthy people.

## 7 Conclusion

Since choroidal vessels are small targets, long-range dependencies need to be considered, therefore, we developed a two-branch fine-grained feature extraction module that can mix the long-range information extracted by TransFormer with the local information extracted by convolution in parallel, introducing information exchange between the two branches. To address the problem of low contrast and blurred boundaries of choroidal vessels in OCT images, we developed a large kernel and multi-scale attention module, which can improve the features of the target area through multi-scale convolution kernels, channel mixing and feature refinement.

The proposed MFGNet was trained on highly myopic and healthy OCT samples and subjected to rigorous evaluation in order to ascertain its efficacy. The experimental results demonstrate that our MFGNet is superior to other competing approaches in both choroidal sublayer and large vessel segmentation. Subsequently, the large vessel segmentation results obtained from the MFGNet model, which had undergone extensive training, were employed in the 3D reconstruction process for further analysis. The 3D reconstruction results of the large vessels demonstrate a high degree of agreement with the *en face* images of the choroidal Haller layer, particularly in terms of the morphology of the visible primary vessels. Furthermore, three vascular-related parameters were calculated based on the 3D reconstruction, and a comparison was made between high myopia and healthy controls. The statistical analysis revealed significant differences between highly myopic and healthy subjects in terms of the 3D fractal dimension and 3D vascular density of large vessels. These findings illustrate the significant clinical potential of further investigation into choroidal vascular analysis.

## Data Availability

Publicly available datasets were analyzed in this study. This data can be found here: https://imed.nimte.ac.cn/OCHID-plus.html
